# Gender, smoking, and tobacco cessation with pharmacological treatment in a cluster randomized clinical trial

**DOI:** 10.18332/tid/177260

**Published:** 2024-02-15

**Authors:** César Minué-Lorenzo, Eduardo Olano-Espinosa, María Minué-Estirado, Jose-María Vizcaíno-Sánchez, Francisco Camarelles-Guillem, José-Antonio Granados-Garrido, Margarita Ruiz-Pacheco, María Isabel Gámez-Cabero, Francisco Javier Martínez-Suberviola, Encarnación Serrano-Serrano, Isabel Del Cura-González

**Affiliations:** 1 Centro de Salud Perales del Río, Dirección Asistencial Centro, Servicio Madrileño de Salud, Madrid, España; 2 Red de Investigación en Cronicidad, Atención Primaria y Prevención y Promoción de la Salud, Madrid, España; 3 Instituto de Investigación Sanitaria Hospital Doce de Octubre (i+12), Madrid, España; 4 Research Network on Preventive Activities and Health Promotion, Madrid, Spain; 5 Centro de Salud Los Castillos, Dirección Asistencial Oeste, Servicio Madrileño de Salud, Madrid, España; 6 Centro de Salud José María Llanos, Dirección Asistencial Este, Servicio Madrileño de Salud, Madrid, España; 7 Instituto de Investigación Sanitaria Gregorio Marañón, Madrid, España; 8 Centro de Salud Fuentelarreina, Dirección Asistencial Norte, Servicio Madrileño de Salud, Madrid, España; 9 Centro de Salud Infanta Mercedes, Dirección Asistencial Norte, Servicio Madrileño de Salud, Madrid, España; 10 Centro de Salud Guayaba, Dirección Asistencial Centro, Servicio Madrileño de Salud, Madrid, España; 11 Centro de Salud Algete, Dirección Asistencial Norte, Servicio Madrileño de Salud, Madrid, España; 12 Centro de Salud Majadahonda Valle de la Oliva, Dirección Asistencial Noroeste, Servicio Madrileño de Salud, Madrid, España; 13 Centro de Salud Los Fresnos, Dirección Asistencial Este, Servicio Madrileño de Salud, Madrid, España; 14 Unidad de Investigación, Gerencia Asistencial de Atención Primaria, Servicio Madrileño de Salud, Madrid, España; 15 Red de Investigación Servicios de Salud en enfermedades crónicas, REDISSEC, Madrid, España

**Keywords:** Spain, healthcare financing, primary healthcare, smoking cessation, gender studies

## Abstract

**INTRODUCTION:**

Whether men find it easier to quit smoking than women is still controversial. Different studies have reported that the efficacy of pharmacological treatments could be different between men and women. This study conducted a secondary analysis of ‘Subsidized pharmacological treatment for smoking cessation by the Spanish public health system’ (FTFT-AP study) to evaluate the effectiveness of a drug-funded intervention for smoking cessation by gender.

**METHODS:**

A pragmatic randomized clinical trial by clusters was used. The population included smokers aged ≥18 years, smoking >10 cigarettes per day, randomly assigned to an intervention group receiving regular practice and financed pharmacological treatment, or to a control group receiving only regular practice. The main outcome was continued abstinence at 12 months, self-reported and validated with CO-oximetry. The percentage, with 95% confidence intervals, of continued abstinence was compared between both groups at 12 months post-intervention, by gender and the pharmacological treatment used. Multilevel logistic regression analysis was performed.

**RESULTS:**

A total of 1154 patients from 29 healthcare centers were included. The average age was 46 years (SD=11.78) and 51.7% were men. Overall, the self-reported abstinence at 12 months was 11.1% (62) in women and 15.7% (93) in men (AOR=1.4; 95% CI: 1.0–2.0), and abstinence validated by CO-oximetry was 4.6% (26) and 5.9% (35) in women and men, respectively (OR=1.3; 95% CI: 0.7–2.2). In the group of smokers receiving nicotine replacement treatment, self-reported abstinence was higher in men compared to women (29.5% vs 13.5%, OR=2.7; 95% CI: 1.3–5.8).

**CONCLUSIONS:**

The effectiveness of a drug-financed intervention for smoking cessation was greater in men, who also showed better results in self-reported abstinence with nicotine replacement treatment.

## INTRODUCTION

Smoking is the leading cause of preventable disease and death in the world. In 2019, tobacco consumption caused more than 8.67 million deaths worldwide, 2.14 million of which were women; in Europe, 1.2 million men and half a million women died for the same reason^1^. The annual mortality rate in Spain due to tobacco consumption in 2017 was 53825 people, of which 45520 were men and 8305 were women^2^.

Although the prevalence of people who smoke daily has decreased in recent years to 19.6% of the worldwide population, the total number of smokers is still very high, with at least 940 million men and 193 million women considered smokers^1^. According to Eurobarometer^3^, 23% of the European Union population smokes (26% of men versus 21% of women). In Spain, almost 20% of the population aged >15 years smokes daily (23.3% of men and 16.4% of women)^4^. The distribution by age shows a higher prevalence among men, although the difference between genders is smaller at younger ages. In Spain, the proportion of men who smoke daily has fallen by 18 points over the last 25 years, versus a decrease of only 5 points among women. Therefore, the difference in the proportion of smokers of both genders has decreased from 32% reported 30 years ago to only 7 points. As gender inequality has declined in Spain over the last 50 years, the prevalence of tobacco consumption among women, which was initially much lower, has converged over time to that of men^5^. Some surveys show this gender gap has even been reversed for certain age groups. The study ESTUDES 2021^6^ shows that both daily and last-30-day consumption is higher in girls for most ages (between 14 and 18 years).

Smoking increases the risk of numerous diseases in both men and women, and associations are continuously established with other conditions with which it has not been associated to date^7^. A meta-analysis that analyzed the overall risk of disease by sex found that this risk is higher in women and that this difference among genders increases with greater tobacco consumption^8^. The risk of coronary heart disease is 25% higher in female versus male smokers^9^. Women may also be at increased risk for some specific types of cancer, such as colorectal or bladder cancer^10^. In other cases, such as chronic obstructive pulmonary disease (COPD), susceptibility to developing the disease for equal levels of exposure appears to be greater in women, in addition to being frequently associated with under-diagnosis and under-treatment, which has been identified as gender influence as it is considered a ‘men’s disease’^11^. Lung cancer, the prototypical tobacco-related disease, is currently the leading cause of cancer mortality in women in the USA as well as in some European countries^12^.

On the other hand, tobacco consumption in women is associated with alterations in menstrual function and pregnancy, potential complications with the use of oral contraceptives, and increased risk of cervical cancer or breast cancer during post-menopause^13^.

The FTFT-AP study^14^ aimed to assess the effectiveness of financing pharmacological treatment for smoking cessation in the adult population in the National Health System (NHS) primary care setting. The study design, which considered the same intervention in men and women, found a difference by sex that warrants an exhaustive secondary effectiveness analysis by gender. Progressing in this regard could help develop interventions that favor equal opportunities for smoking cessation. This study aimed to evaluate the effectiveness of a drug-funded intervention for smoking cessation by gender.

## METHODS

The study used a pragmatic, controlled, paralleled, cluster-randomized clinical trial. The study was approved by the Ethics Committee for Clinical Research of Hospital Doce de Octubre de Madrid, reference number 06/226. The study followed the ethical principles originating in or derived from the Declaration of Helsinki and complies with the Good Clinical Practice Guidelines by the International Conference on Harmonization. Written informed consent for participation was obtained from all the included subjects.

Twenty-nine health centers in the Autonomous Community of Madrid participated in the study. The randomization unit was the healthcare center, and the analysis unit was the smoking patient. The study sample was the 1154 patients participating in the original project^14^, aged >18 years, smoking >10 cigarettes per day, who attended the healthcare center for any reason, and who were questioned about their tobacco consumption by their doctor or nurse. Both groups were offered the usual clinical management at the health center, consisting of a cognitive-behavioral intervention. The pharmacological treatment was fully financed in the intervention group, whereas it was at the patient’s expense in the control group. The professional chose the type of drug based on the patient’s preferences.

The variables recorded at the baseline were age, gender, education level, income level, daily cigarette consumption, yearly package consumption, score on the Fagerström test of nicotine dependence (FTND)^15^, number of previous quit attempts, change stage, and former use of pharmacological treatment. The main outcome variables were continued abstinence after 12 months according to Russell’s criteria^16^ (self-report of smoking not more than five cigarettes from the start of the abstinence period at the final follow-up), and abstinence biochemically validated with CO-oximetry. The secondary variables were utilization and type of pharmacological treatment, namely nicotine replacement therapy (NRT), bupropion, or varenicline. Data were obtained from the electronic medical records. Whenever the required information could not be retrieved from such records, the patient was contacted by phone to confirm or not abstinence. Patients who confirmed abstinence were scheduled for CO-oximetry testing. The cut-off point for validation was set at <7 ppm. An intention-to-treat analysis was performed.

### Statistical analysis

Qualitative variables were described by their frequencies and percentages and quantitative variables by their means and standard deviation (SD). Intergroup comparisons were performed. For the bivariate analysis, the chi-squared test was used to compare qualitative variables and Student’s t-test for quantitative variables.

The intergroup difference in the rate of self-reported abstinence and chemically validated abstinence after one year was calculated and disaggregated by sex. The difference in the rate of self-reported abstinence and chemically validated abstinence between men and women was also estimated with their 95% confidence interval (CI). The use of pharmacological treatment and the efficacy of each treatment (NRT, bupropion, or varenicline) were measured for men and women. A multilevel logistic regression model (taking into account sampling by clusters) was constructed for men and another for women^14^. STATA 14 software was used for all analyses.

## RESULTS

**Figure 1:**
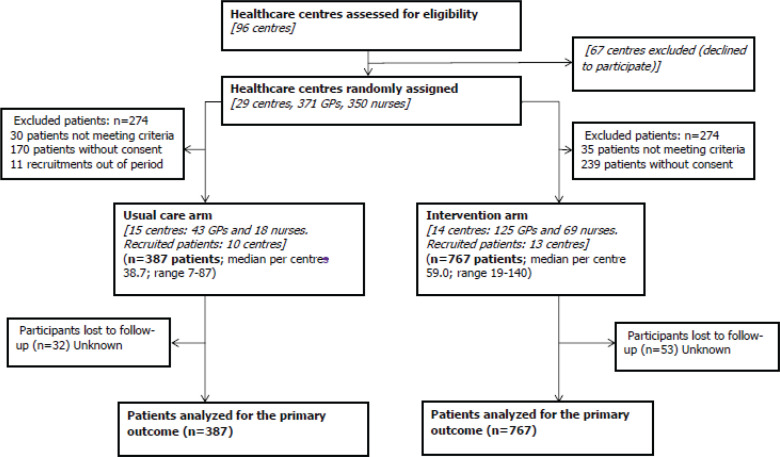
Flow chart of participant selection

A total of 255 professionals from 23 health centers participated in the trial. The study sample included 1154 patients, 387 in the control group and 767 in the intervention group. The average age was 46 years, and the sex distribution was 593 men and 560 women. Figure 1 shows the flow diagram of the study participant selection process.

Baseline differences were found in the sex distribution, with a higher number of women in the control group (55% vs 45%) and in the number of cigarettes and pack-years index, as well as in the income level, which was higher in men. No significant differences were found in the rest of the variables, including the Fagerström test for nicotine dependence (FTND) (Table 1).

**Table 1:** Patient’s characteristics by sex

*Characteristics*	*Overall (N=1153) n (%)*	*Men (N=593) n (%)*	*Women (N=560) n (%)*	*p*
**Control group**	387 (33.6)	174 (29.3)	213 (38.0)	0.002
**Intervention group**	766 (66.4)	419 (70.7)	347 (62.0)	0.002
**Age** (years), mean (SD)	46.0 (11.8)	45.9 (12.5)	46.2 (11.0)	0.663
**Education level**				
Uneducated	25 (2.5)	14 (2.8)	11 (2.3)	0.811
Basic education	326 (32.9)	173 (34.0)	153 (31.7)	
Secondary education	386 (39.0)	196 (38.5)	190 (39.4)	
Higher education	254 (25.6)	126 (24.8)	128 (26.6)	
**Yearly income level** (€)				
<26000	668 (69.6)	307 (46.0)	361 (54.0)	0.001
≥26000	291 (30.3)	183 (62.9)	108 (37.1)	
**Cigarettes/day,** mean (SD)	22.0 (9.5)	22.8 (10.2)	21.0 (8.6)	0.001
**Years smoking,** mean (SD)	26.5 (11.9)	27.0 (12.9)	25.9 (10.7)	0.146
**Pack-years,** mean (SD)	29.6 (19.9)	31.1 (21.7)	28.0 (17.7)	0.008
**Previous quit attempts,** mean (SD)	2.3 (3.2)	2.5 (3.7)	2.1 (2.4)	0.063
**FTND score^a^,** mean (SD)	5.4 (2.2)	5.5 (2.3)	5.4 (2.2)	0.598
**Pharmacological treatment**				
NRT	177 (15.4)	88 (14.8)	89 (15.9)	0.679
Bupropion	180 (15.6)	92 (15.5)	88 (15.7)	0.990
Varenicline	248 (21.5)	130 (21.9)	118 (21.1)	0.780

a FTND: Fagerström test for nicotine dependence.

Overall, 11.1% of the included women (62) reported having quit smoking versus 15.7% of men (93) (Table 2). Self-reported continued abstinence was higher in men, with an AOR of 1.4 (95% CI: 1.0–2.0). The difference in abstinence with CO-oximetry validation was not statistically significant, with 35 men (5.9%) who quit smoking versus 26 women (4.6%) (AOR=1.3; 95% CI: 0.7–2.2). The number of participants interested in making an attempt to quit was similar in men and women, as was the use of pharmacological treatment. The use of pharmacological treatment improved abstinence rates (Table 2).

**Table 2:** Continuous abstinence, use of pharmacological treatment, and attempts to quit smoking, by sex

*Variables*	*Men n (%)*	*Women n (%)*	*OR (95% CI)*	AOR (95% CI)*
Continuous abstinence	93 (15.7)	62 (11.1)	1.5 (1.1–2.1)	1.4 (1.0–2.0)
Validated abstinence	35 (5.9)	26 (4.6)	1.3 (0.8–2.2)	1.3 (0.7–2.2)
Pharmacological treatment	298 (50.3)	285 (50.9)	1 (0.8–1.2)	0.9 (0.7–1.1)
Quit attempts	327 (55.1)	315 (56.2)	0.9 (0.8–1.2)	0.9 (0.7–1.1)

* AOR: adjusted odds ratio; adjusted by arm (control or intervention).

**Figure 2:**
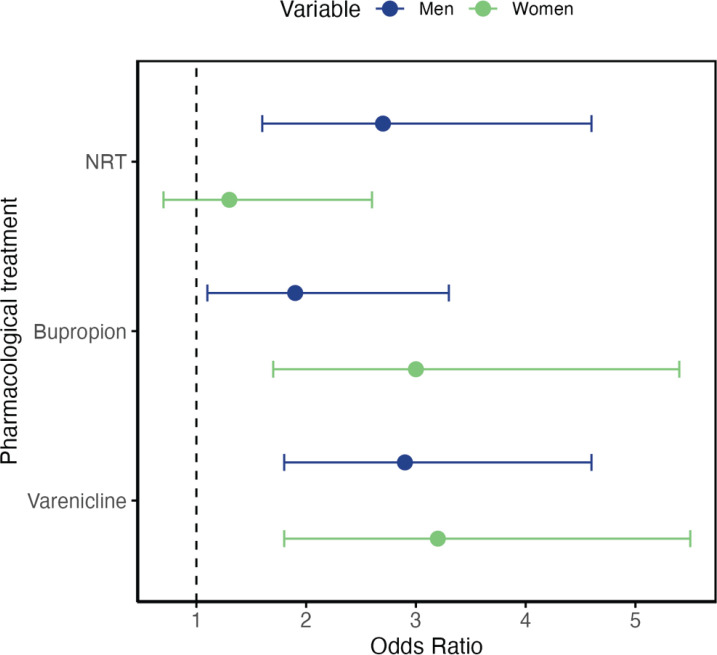
Type of pharmacological treatment and continuous abstinence at one year, by sex

Each of the three available treatments was also associated with a higher abstinence rate on its own. NRT (OR=2; 95% CI: 1.3–3), bupropion (OR=2.3; 95% CI: 1.5–3.4), and varenicline (OR=3; 95% CI: 2.1–4.3). However, while the last two drugs were effective in both men and women, 13.5% of women who used NRT quit smoking compared to 10.6% of women who did not use NRT (OR=1.3; 95% CI: 0.7–2.6). For men, 29.5% of NRT users achieved abstinence compared to 13.3% of non-users (OR=2.7; 95% CI: 1.6–4.6). In the group of smokers receiving nicotine replacement therapy, the self-reported abstinence was higher in men compared to women [29.5% versus 13.5%, respectively; (OR=2.7; 95% CI: 1.3–5.8)] (Figure 2). In terms of self-reported abstinence, 12.7% of the women and 17.7% of the men reported quitting smoking in the intervention group versus 8.5% of the women and 10.9% of the men in the control group. The difference in abstinence between genders was not significant in the control group (p=0.4) but did reach statistical significance in the intervention group (p=0.04).

Higher education level or income level (≥ €26000 per year) increased abstinence in both genders, although without reaching statistical significance. Smoking cessation was greater in women with a higher income (14.8% vs 9.4%) and also in men (19.7% versus 14.7%).

In estimating the total effect of the intervention, adjusted for sex, we find that men were more likely to achieve abstinence at one year than women (Table 3).

**Table 3:** Total effect of the intervention on abstinence, by sex

	Global model*			*Men model*			*Women model*		
	*OR*	*95% CI*	*p*	*OR*	*95% CI*	*p*	*OR*	*95% CI*	*p*
Intervention (yes/no)	1.77	1.09–2.88	0.02	1.74	1.02–2.99	0.04	1.72	0.83–3.56	0.14
Sex (woman)	1.44	1.02–2.05	0.03						

* AIC=904.6225. BIC=924.823.

Four per cent of the abstinence variability per year is explained by clusters (HCCs). The MOR (median odds ratio) between centers was 1.4, which can be interpreted as the increase in risk (median) that an individual would have if they were moved from one center to another with a higher risk.

## DISCUSSION

This study is part of the FTFT-AP clinical trial designed to determine the effect of financing pharmacological treatment for smoking cessation in the primary care setting. A secondary analysis of this study was conducted to analyze effectiveness by sex.

The results suggest that the intervention (funding drug treatment) may be more effective in men, although at the limit of statistical significance. Differences were found in the allocation of women to the groups (greater number of women in the control group) and in the fact that, overall, the proportion of men who succeeded in quitting smoking was greater, despite the fact that the baseline tobacco consumption was higher in men. In the branch of the intervention group receiving NRT, statistically significant differences in abstinence at 12 months were found only in men.

These results are in agreement with those of other studies. Men successfully quit smoking in a higher proportion than women. Continued self-reported abstinence after one year was 15.7% in men and 11.1% in women. After adjusting the logistic regression model to take into account the effect of the intervention in the clinical trial (in which financed pharmacological treatment for smoking cessation was offered), the OR remained significant. An extensive review by Smith^17^ found that the proportion of women who discontinue smoking is significantly lower compared to men in clinical trials, with only one of 37 efficacy trials and only one of 79 effectiveness trials showing better results in women. The evidence is less consistent in observational studies: most prospective studies obtain similar results in both genders (31 of 46 comparisons), with five reporting better results in women and 10 in men. Earlier studies tend to find more differences in abstinence outcomes than more recent ones. Different sociocultural factors and changes over time may play an important role in the evolution of these differences.

In our setting, three studies^18-20^, all of them performed after the year 2000, obtained better outcomes in men. The ISTAPS study, a cluster randomized clinical trial that was conducted in health centers in 13 administrative regions in Spain, analyzed gender as a predictor of cessation in an intervention based on the Transtheoretical Model of Change. Over 2800 subjects participated in the trial, half of which were women^21^. There were no statistically significant differences in abstinence rates between men and women.

In the present study, NRT was less effective in women than in men, a difference that was at the borderline of statistical significance. Some studies indicate that NRT may be less effective in women^22^. In a meta-analysis examining 14 clinical trials, nicotine patches were effective in 20.1% of men and 14.7% of women compared with 10.8% and 10.1%, respectively, receiving a placebo. The OR was 1.61 in women and 2.2 in men, and the OR for the interaction was 1.40 (95% CI: 1.02–1.93)^23^.

An analysis of real-life data^24^ showed that NRT was more effective than any other treatment in men but not women, while varenicline was superior to NRT in women but not men. A network meta-analysis^25^ in the general population found that varenicline was superior to NRT and bupropion, with no differences between the last two. In women, varenicline was superior to bupropion and NRT, and in men the effectiveness of the three treatments was similar.

Studies that analyze the effect of funding drugs for smoking cessation on abstinence do not offer data disaggregated by gender. In our study, medication funding did not significantly increase abstinence in women, but it did in men. The difference between the two groups was not statistically significant, suggesting a lack of power to support this finding. The observed differences may be explained by the different ways in which women and men relate to tobacco. Men tend to start smoking earlier, consume more cigarettes per day, and have a greater dependence on tobacco^26^. In this study, the FTND score was similar in men and women. Studies investigating the relationship of tobacco consumption with sex/gender are very heterogeneous in their methodology, approach, context, and findings. Although many studies indicate greater difficulty for women to discontinue smoking, the role of sex, gender, and their interrelationship in the results remains unclear, as these are influenced by the specific context and the interaction of sex/gender with other axes of inequality^17^.

Women’s smoking behavior has been suggested to be more related to factors of relieving negative emotions or sensory stimuli, while men’s smoking behavior is more related to nicotine reward^27^. However, the outcomes reported in the literature are contradictory, and several real-life ecological studies do not find gender differences in terms of exposure to stimuli, stressful situations, or social aspects. Men may smoke more in conjunction with alcohol or food consumption, or in the presence of other smokers^28^. Some authors postulate that mood, distress, stress, and anxiety are predictors of relapse in women, while the main predictor in men is craving^13^. The differentiation of biological aspects more strongly associated with sex^22,29^ from others related to gender is especially complex in the case of addictions and has not been sufficiently investigated. Gender roles could explain differences in tobacco use between men and women, as well as their behavior in terms of cessation attempts and response to pharmacological treatment. This relationship remains understudied in the scientific literature^30^. Biomedical approaches predominate when assessing the relationship of women with tobacco use, devoid of a social approach that takes into consideration female and male gender roles, their identities, and the relationships between them^11^. The effectiveness of specific approaches for women has rarely been investigated^30^. Interventions for smoking cessation do not usually involve different strategies for men and women, except in the case of pregnant women^31^ where, on many occasions, a guilt-based approach can reduce the probability of quitting, in addition to perpetuating gender inequalities. Some interventions in women focused on concerns about weight gain being helpful, although the evidence is not definitive^32^.

### Strengths and limitations

This was a cluster trial in which cluster randomization was performed before the recruitment of participants, which could have led to a selection bias. As a result, the centers in the intervention group recruited more patients in a shorter period than those in the control group. In the latter, more women were differentially included. A bias by professionals in offering participation to men cannot be ruled out, due to the historical connection of men with tobacco use and dependence. The gender of the prescribing professionals and its possible influence has not been analyzed either. The estimated sample size was not achieved for several reasons^14^. This led to insufficient power to conclusively demonstrate some of the results. Losses to follow-up, which are common in smoking trials, were also numerous in this study, with similar figures in both genders. At the time of designing the study, the gender perspective was not taken into account. Therefore, following the recommendations of clinical practice guidelines, the assessed intervention was common to both men and women. Most clinical practice guidelines for the treatment of tobacco dependence do not consider gender, despite evidence of the influence of gender and its intersection with culture, age, or social class on tobacco consumption and the way of relating to tobacco. There is no agreement on the use of validated tools to evaluate gender. In addition to differentiating sex and gender among the sociodemographic data, tools are necessary to assess the role of cultural, institutional, and family norms and power relations^33^ among individuals of different gender identities^34^.

One of the strengths of the study is its pragmatic nature. The smokers were treated by their assigned health professionals when they attended their usual consultations, with no other conditioning factors. The inclusion criteria were patients aged >18 years for whom tobacco use was addressed during the consultation, and the exclusion criterion was no indication for pharmacological treatment^35^. The interventions were conducted under conditions of real clinical practice in the Madrid primary care setting and were delivered flexibly, without formal protocolized visits. However, the results are not generalizable to other countries and other contexts.

### Applicability to clinical practice and research

Research on the effectiveness of different approaches to smoking cessation, taking gender into consideration, remains limited^30^. Proposals for approaches specifically targeting women tend to focus on biological and reproductive health components (pregnancy, fertility, hormonal status, and other medical conditions). Interventions that consider women’s concerns about weight gain have shown promising results, but there is no clear evidence at this time that specific interventions yield better results^32^. Providing evidence with mixed quantitative and qualitative methodologies is warranted, incorporating epidemiological and social science research to account for the social determinants of tobacco addiction. It is necessary to deepen the study of the differences in consumption patterns depending on sex and gender and propose gender-specific interventions that move towards equity, instead of perpetuating gender stereotypes (motivating women to quit with arguments based on beauty, etc.)^30^. Such proposals should be specific for the addressed culture and consider the intersection of gender with other axes of inequality that are part of the social determinants that condition people’s health^36^.

## CONCLUSIONS

The present study found that the abstinence rate at one year was significantly higher among male smokers than among women, among which NRT seems to be less effective. These differences must be taken into consideration when designing strategies to address tobacco consumption in the general smoking population. Research with specific designs should be promoted, incorporating cultural competence and considering the intersection of gender with other axes of inequality.

## Data Availability

The data supporting this research are available from the authors on reasonable request.
